# Origin of Infrared Light Modulation in Reflectance-Mode Photoplethysmography

**DOI:** 10.1371/journal.pone.0165413

**Published:** 2016-10-21

**Authors:** Igor S. Sidorov, Roman V. Romashko, Vasily T. Koval, Rashid Giniatullin, Alexei A. Kamshilin

**Affiliations:** 1 R&D Center for Laser Physics, ITMO University, St. Petersburg, 197101, Russia; 2 Precision Optical Measurement Techniques Department, Institute for Automation and Control Processes of FEB RAS, 5 Radio St., Vladivostok, 690041, Russia; 3 Far Eastern Federal University, 8 Sukhanova St., Vladivostok, 690900, Russia; 4 Federal State Institution "1477 Navy Clinical Hospital", Vladivostok, 690005, Russia; 5 Department of Neurobiology, University of Eastern Finland, 70210 Kuopio, Finland; 6 Laboratory of Neurobiology, Kazan Federal University, 420000 Kazan, Russia; 7 Department of Computer Photonics and Videomatics, ITMO University, St. Petersburg, 197101, Russia; Vanderbilt University Medical Center, UNITED STATES

## Abstract

We recently pointed out the important role of dermis deformation by pulsating arterial pressure in the formation of a photoplethysmographic signal at green light. The aim of this study was to explore the role of this novel finding in near-infrared (NIR) light. A light-emitting diode (LED)-based imaging photoplethysmography (IPPG) system was used to detect spatial distribution of blood pulsations under frame-to-frame switching green and NIR illumination in the palms of 34 healthy individuals. We observed a significant increase of light-intensity modulation at the heartbeat frequency for both illuminating wavelengths after a palm was contacted with a glass plate. Strong positive correlation between data measured at green and NIR light was found, suggesting that the same signal was read independently from the depth of penetration. Analysis of the data shows that an essential part of remitted NIR light is modulated in time as a result of elastic deformations of dermis caused by variable blood pressure in the arteries. Our observations suggest that in contrast with the classical model, photoplethysmographic waveform originates from the modulation of the density of capillaries caused by the variable pressure applied to the skin from large blood vessels. Particularly, beat-to-beat transmural pressure in arteries compresses/decompresses the dermis and deforms its connective-tissue components, thus affecting the distance between the capillaries, which results in the modulation of absorption and scattering coefficients of both green and NIR light. These findings are important for the correct interpretation of this widely used medical technique, which may have novel applications in diagnosis and treatment monitoring of aging and skin diseases.

## Introduction

Classically and commonly used in clinical practice, photoplethysmography (PPG) is based on a contact single point detection of the amount of light either transmitted or reflected to the photodetector [1]. Despite the wide range of applications, interpretation of the PPG signal is still a matter of debate. Conventional PPG sensors typically operate with infrared light, which penetrates relatively deep into biological tissue and may interact with the pulsatile blood of the arteries. It is well known that the light wavelength determines the depth of light penetration into skin [2]. Therefore, it is expected that infrared illumination widely used in medical applications, such as functional near-infrared (NIR) spectroscopy, should provide more optimal signal detection than, for instance, green light with limited penetration capacity.

However, in the last decade, several research groups developed imaging photoplethysmography (IPPG) systems [3–8], which mainly operate with green light. In contrast with conventional PPG sensors requiring tissue contact, IPPG in the reflectance mode could provide contactless vital-sign monitoring. In addition, IPPG systems have the important advantage of offering detailed spatial information simultaneously from different places, thus allowing mapping of physiological parameters. Recently, a modified IPPG approach referred to as blood pulsation imaging (BPI), which allows mapping both the amplitude and relative phase of blood pulsations, was proposed by our group [9]. Using this method, we found new biomarkers of a migraine in the facial area, indicating the usefulness of this method for clinical practice and opening new perspectives for novel applications [10]. These systems are very simple in their implementation and cost-efficient, because they consist only of using a conventional camera and an illuminating source. Moreover, several groups recently reported on the development of IPPG systems operating with ambient light [6–8]. It is worth noting that there is a message from all papers devoted to IPPG systems operating with the ambient light stating that the highest signal-to-noise ratio (SNR) is observed in the green channel of conventional RGB cameras used in their systems. Researchers working with the contact PPG sensors have also reported on the better performance of the sensors using green light for a subject’s illumination [11]. However, direct comparison between green and infrared illumination in IPPG was not reported so far.

In this work, we carried out a direct experimental comparison of IPPG-system performance at green and NIR illumination. Moreover, considering recent observations of the strong influence of skin-contact status on the green-light interaction with dermis [12], we measured the effect of a skin-glass contact in both wavelengths simultaneously. Contrary to expectation, illumination with green light provides superior signal readouts compared with infrared light. We have also observed a significant increase of the PPG-waveform amplitude at both wavelengths after the skin was brought into contact with glass. Although our group recently reported such an increase at green illumination [12,13], the increase of PPG amplitude at infrared light was observed for the first time. Analysis of the observations suggests that the origin of NIR light modulation is the elastic deformation of the dermis. This finding indicates that correct modeling of light interaction (both visible and NIR) with living tissue should take into account compression of the dermis by transmural arterial pressure that varies in time.

## Subjects and Methods

### Participants

Measurements were carried out with 34 healthy subjects (25 men and 9 women). The age of the subjects varied from 18 to 66 years. Persons with any neurologic, cardiovascular, or skin disease were excluded from the study. Experiments were performed in the Federal State Institution “1477 Navy Clinical Hospital” of the Ministry of Defense of the Russian Federation, Vladivostok, Russia. This study was conducted in accordance with the standards of application of new medical techniques laid down by Order of the Ministry of Health of the Russian Federation No. 25 on February 16, 1994. The Research Ethics Committee of the 1477 Navy Hospital (Vladivostok) approved the study plan on February 9, 2015. Ethical approval was obtained before the study. All subjects gave their written informed consent to participate in the experiment.

### Experimental setup

**Fig 1:**
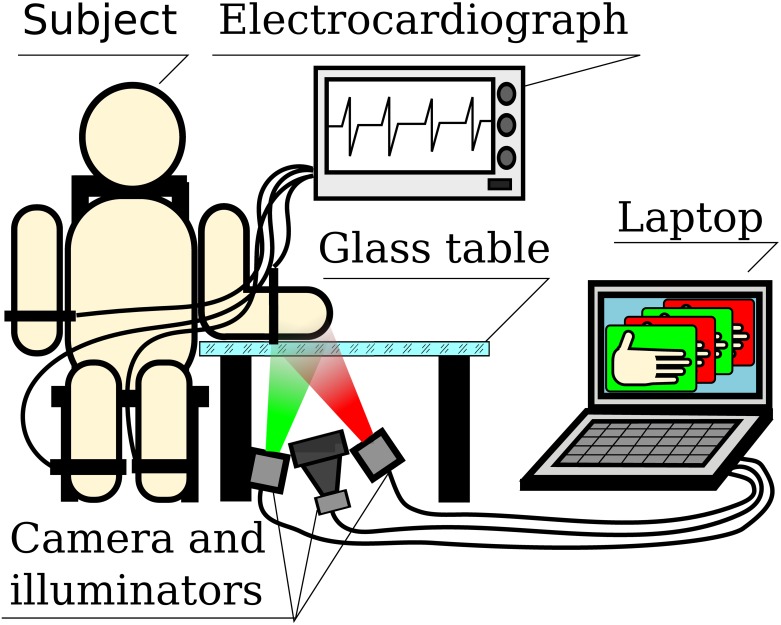
Simplified layout of the experiment. Video of subject’s palm and electrocardiogram were simultaneously recorded. Green illumination of the palm was switched to the NIR and vice versa in a frame-to-frame regime.

For data acquisition, we used a custom-made IPPG system allowing us to illuminate a subject by light at two different wavelengths by switching them at each frame. A layout of the experiment is shown in Fig 1. The IPPG system consisted of the illuminator and digital camera. Four light-emitting diodes (LEDs) were used: two operating at a wavelength of 525 nm (green) and two at 810 nm (NIR). The optical power of green and NIR LEDs was 30 mW and 70 mW, respectively. Their spectral bandwidths were 40 nm and 60 nm, respectively. Each pair of LEDs provided almost uniform illumination of the area of 25 cm × 40 cm at the distance of 70 cm. Before the experiment for each subject, we measured the average pixel value over the palm area in the recorded video frames separately for green and NIR illumination. Then the electric current of either green or NIR LEDs was adjusted so that the mean difference of camera responses at both wavelengths did not exceed 20%. The light incident angle for both wavelength did not exceed 15 degrees while the camera was situated so that the specular reflection from the glass was near the field of view but out of it. The distance between the camera lens and the place of measurements was 110 cm. The illuminator was synchronized with the camera. A trigger pulse generated by the camera at the beginning of each frame switched on either green or NIR LEDs. Thus, each even camera frame was recorded when the palm is illuminated by green LEDs but each odd frame—by NIR LEDs. Additionally, to exclude the influence of direct reflections from interfaces air/skin, air/glass, and glass/skin, we have used a pair of crossed polarizers. One of these polarizers was situated in front of the illuminator, and another was attached to the camera lens. The extinction ratio of the linear polarizing films was 1:90 and 1:18 at the green and NIR lights, respectively.

A digital black-and-white complementary metal-oxide semiconductor (CMOS) camera (GigE uEye UI-5220SE of Imaging Development Systems GmbH) provided video recordings of the synchronously illuminated area. To increase the spectral sensitivity of the camera to NIR light, the infrared-protection filter was changed for a normal glass K8. The area under study included the subject’s palm and a part of the wrist. The camera was used in conjunction with a lens (18–108 mm focal length, Canon, Japan). Focused images of the illuminated area were recorded with pixel resolution of 752 × 480 at the frequency of 60 frames per second (fps) during 24 seconds. Owing to synchronization of the illuminator and camera, which provides illumination of each second frame at the same wavelength, we obtained two 24s videos: one for the green light and another for NIR light. The videos were shifted in the time domain in respect to each other for the duration of one frame, which is 16.7 ms. The effective frame rate of the video for each wavelength was equal to 30 fps. The measurements were performed in the dark room to exclude the influence of the artificial illumination. All video frames were saved on a laptop computer in the portable network graphics (PNG) format. Our preliminary study [12] had shown that there was no information losses concerning the temporal modulation of the pixel value at the heartbeat frequency if we use PNG format instead of uncompressed bitmap-image format, but the frames in the former format occupied less computer memory. For each subject, simultaneously with video, we recorded an electrocardiogram (ECG) by means of digital electrocardiograph (Fukuda CardiMax FX-7102). ECG was recorded using disposable Ag/AgCl electrodes attached to the left and right wrists with the reference electrodes on the legs.

### Experimental procedure

Each subject was kept in a quiet laboratory room in a seated position before video and ECG recordings. During the relaxation period of 15 minutes, we measured the subjects’ blood pressure, and the subjects completed a questionnaire regarding his or her physical status. Thereafter, the subject was asked to sit comfortably while placing his hand on the glass table, as shown in Fig 1. Our IPPG system was placed under the table to provide capturing palm images through the glass. First, we recorded 24-second video without contact between the palm and the glass (the wrist and fingers were on two soft supports in this case). Second video of the same duration was recorded when the subject put his or her hand onto the glass with an additional weight of 2.0 kg over the hand. Individual features of the palm’s shape lead to uneven distribution of the external pressure, affecting the skin in different areas of the contact. Mean pressure in the contact was estimated for every subject by dividing the preliminarily measured force (hand’s weight including additional load) on the contact area, which was obtained by analyzing one of the raw images. The mean contact pressure varied from 27 to 55 mmHg with an average of 35 mmHg.

Assuming that fast transient processes (which occur faster than 16.7 ms) in the blood perfusion are small compared with the main cardiac activity with the period of about 1 second, we picked up almost the same information about the state of the cardiovascular system during both green and NIR illumination because of fast switching between these wavelengths.

### Data processing

Data processing was done offline by using custom software implemented in the MATLAB^®^ platform. In a core of the algorithm, we calculated spatial distribution of the amplitude of PPG-signal pulsations over the palm area. In contrast with our previous BPI systems [5,9,14], here we used a more straightforward algorithm for the calculation of the pulsation amplitude. We considered a frame-to-frame evolution of the pixel values as a PPG-waveform. The duration of each cardiac cycle was estimated from the delay between two consecutive R-peaks in ECG data by using a custom algorithm of R-peaks detection. The positions of the R-peaks in the time scale were considered as cardiac-cycle borders, which were used for calculation of the signal pulsations. The amplitude of PPG-signal pulsations was taken as an AC-to-DC ratio in which AC and DC refer to the peak-to-peak value and the mean value of the PPG waveform in every cardiac cycle, respectively. We calculated PPG waveforms in the resized frames obtained from the initial video frames by averaging pixel values within non-overlapping areas of 4 × 4 pixels. Such a resizing allowed us to increase SNR of the PPG signals at the expense of diminishing the spatial resolution of images down to 188 × 120 pixels. Amplitude maps of the PPG signal were calculated for each pixel of the resized image by averaging in time the AC/DC ratios over all cardiac cycles during 24 seconds. In this way, we composed amplitude maps of PPG waveforms for all four measurements: contact and contactless, and each of them under green and NIR illuminations. The mean DC level of the PPG signal was controlled in each experiment. Under green illumination, the mean DC in the contact experiment was 1.15 ± 0.04 times higher than in the contactless one. The similar contact/noncontact DC-level ratio for NIR illumination was 1.09 ± 0.04. Both ratios changed slightly from subject to subject. An increase of the DC level in the contact conditions relates to a smaller distance between the illuminator and a palm because of 8-mm thick soft supports used in the contactless experiment.

Thereafter, we compared the influence of the skin contact on the PPG amplitude for green and NIR illumination. To this end, we found a point with the maximal amplitude (“hot” spot) for each palm of each subject in the PPG-amplitude maps calculated for the case of the palm in contact with glass. Note that each point in the PPG-amplitude map originates from a 4 × 4 pixel area of the initially recorded frames. Because these frames were recorded when both green and NIR light remitted from a subject’s palm were collected by the same lens into the same photosensitive matrix, we used the same spatial coordinates for both wavelengths while comparing the PPG amplitude as a function of the wavelength. However, comparing contact and contactless amplitude maps, we noticed that the “hot” spots in the contact map anatomically correspond to areas with either moderate or low PPG amplitude in the respective contactless map. To estimate the effect of the contact with glass quantitatively, we first defined the coordinates of the ‘hot’ spot in the contact PPG-amplitude map, and then manually selected an area in the same anatomical place in the contactless map. A manual search of similar anatomical places in different videos is not very accurate. However, approximate estimation is still possible. Error of the amplitude-increase estimations primarily stems from the heterogeneity of PPG amplitude in the contactless experiment. For each subject, we checked variation of the PPG amplitude in the contactless map within the area of 12 × 12 mm^2^ around the selected spot and found that it does not exceed 20% of the amplitude in this spot with the mean deviation of 8% for the whole cohort. Therefore, we considered accuracy of the spot selection acceptable. By finding mean values of PPG-signal amplitude in these spots, we compared the influence of the skin contact on the light modulation for green and NIR illumination.

At first glance, the positions of the “hot” spots at green and NIR light coincide; however, a detailed analysis shows that they may be displaced from one another by one or two pixels, probably due to noise influence. To estimate the spatial correlation between amplitude maps obtained at green and NIR illumination, we compared the coordinates of the “hot” spot maxima for both wavelengths in the glass-contact experiment and calculated the difference as a length of the radius vector.

## Results

### Effect of the skin-glass contact

**Fig 2:**
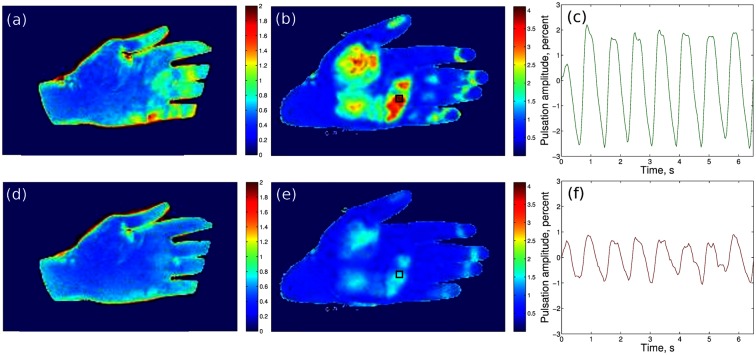
Mapping the PPG-waveform amplitude. Maps obtained at green illumination in the contactless (a) and glass-contact (b) experiments, and at simultaneous NIR illumination in the contactless (d) and glass-contact (e) experiments. Black squares in (b) and (e) show the positions of the “hot” spots in the glass-contact experiment, whereas the graphs (c) and (f) are PPG waveforms calculated in these spots at green and NIR illumination, respectively. Color scales on the right of the maps show the respective PPG amplitude as the AC/DC ratio in percent.

A typical example of the amplitude maps obtained at green illumination in the contactless and glass-contact experiment is shown in Fig 2a and 2b, respectively. PPG-amplitude maps for the same subject’s hand obtained at simultaneous NIR illumination are shown Fig 2d and 2e, respectively. Note that the amplitude scale of pseudocolor maps for the contactless experiment (Fig 2a and 2d) is two times more sensitive than that for the glass-contact experiment (Fig 2b and 2e). Black squares in the maps in Fig 2b and 2e mark the position of the “hot” spots. PPG waveforms in these “hot” spots are shown in Fig 2c and 2f for green and NIR light, respectively. As seen in Fig 2, the amplitude of the PPG signal at green light is twofold larger than at NIR light. It is also seen by the naked eye that spatial distribution of the PPG amplitude at green light correlates well with that at NIR light. Such a behavior was found in all studied subjects.

**Fig 3:**
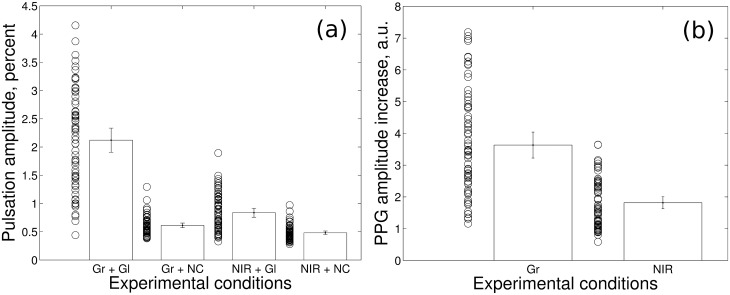
Influence of the skin-glass contact on the PPG amplitude measured at green and NIR wavelengths. (a) Average PPG amplitude measured in “hot” spots in the contact experiment (Gl) and in respective spots in the contactless experiment (NC). Here Gr and NIR stand for green (525 nm) and NIR (810 nm) illumination, respectively. (b) Ratio of the mean amplitude measured in the glass-contact to that in the contactless experiment for both wavelengths. The individual measurements are shown by circles, whereas the bars represent mean values for the whole data sets.

The spatial distribution of the PPG waveform amplitude was calculated for both the left and right hands of each subject. We considered the data obtained from two different hands as statistically independent. Therefore, we performed 68 measurements in total, each of them in both contactless and glass-contact configurations. It is worth noting that the frames at green illumination were captured with the delay of 16.7 ms in respect to the frames at NIR illumination. Thus, two sets of frames (at green and IR illumination) captured information on the arterial blood pulsations almost simultaneously. For each “hot” spot we calculated the mean PPG amplitude when skin contacted the glass plate and when it did not. Individual and average PPG-amplitude values and respective average values were calculated for all four possible combinations (Fig 3a). Fig 3a shows that for both green and NIR illumination, the amplitude of the light modulation at the heartbeat frequency was significantly increased when in contact with glass. However, whereas the mean PPG amplitude increased almost fourfold (from 0.61 ± 0.04% to 2.12 ± 0.21%) at green light, it gained only twofold (from 0.48 ± 0.03% to 0.84 ± 0.08%) at NIR illumination.

The role of skin contact on the PPG signal can be estimated from the ratio of mean PPG amplitudes calculated in the glass-contact to that in the contactless conditions. These ratios measured at green and IR illumination for each palm of each subject are shown in Fig 3b along with the mean values for each wavelength. We found that the contact with glass increased the light modulation up to 7 times at green illumination and up to 3.6 times at NIR illumination. This was a typical result except in a few cases when the average amplitude modulation was less than 0.5%. In average, the PPG amplitude increased by 3.63 ± 0.41 times at green light and by 1.82 ± 0.19 times at NIR light.

### Correlation of the data at green and NIR illumination

**Fig 4:**
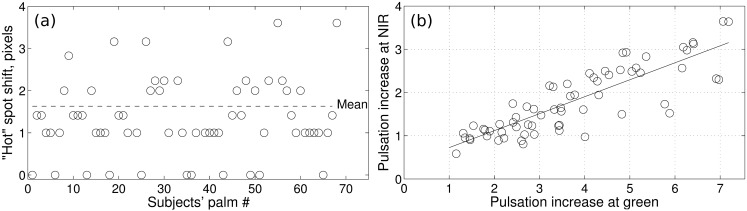
Spatial and amplitude correlation of changes induced by the skin-glass contact at green and NIR illumination. (a) The shift between the centers of the “hot” spots in the amplitude maps found at green and NIR illumination for all studied subjects. (b) Scatter plot of the PPG-amplitude increase due to the contact calculated at green and NIR illumination.

Next, to reveal the mechanism of light interaction with tissue, we estimated the correlation between the changes caused by the contact in both wavelengths. Spatial correlation was estimated as the difference between the position of the “hot” spots found at green and NIR illumination in the glass-contact experiment. This difference is shown in Fig 4a for all subjects in the study. As one can see, “hot” spots in the maps calculated at green illumination are situated near (the mean distance between the centers of the spots is 1.63 pixels, which is approximately 1.5 mm at the palm) the “hot” spots at NIR for most of the measured hands. Another way to demonstrate the correlation between the two data sets is to use a scatter diagram. Such a diagram plotted for the enhancement ratio of the PPG amplitude (due to contact) at NIR as a function of the ratio at green illumination is shown in Fig 4b.

As seen in Fig 4b, there is a strong positive correlation between the data measured at green illumination and that at NIR. In particular, most of the points on the diagram are disposed near the straight line showing the trend of the graph. In other words, the diagram shows that the skin-glass contact increased the modulation amplitude at both green and IR light, but this increase is twice stronger for the green light. We also estimated the correlation between these data sets by using Pearson’s coefficient: r = 0.85 (*P* < 0.001).

## Discussion

The main finding of the current study is that there is a significant increase of PPG amplitude at NIR illumination caused by skin-glass contact. This increase correlates well with the even higher increase at green illumination. According to the classical model of PPG, an AC component of light intensity received by the photodetector in both transmittance and reflectance mode originates from several vascular factors including changes of blood volume, blood-vessel wall movement, and the variable orientation of red blood cells in vessels [15–17]. By the common agreement, the modulation of remitted light at the heartbeat frequency is caused by variation of the tissue optical density, which, in turn, mainly originates from the pulsatile blood volume changes in arteries and arterioles. Blood volume changes in veins and capillaries during a single cardiac cycle are considered insignificant [18]. In other words, in the classical PPG model, the light is modulated in time as a result of direct interaction with the pulsating arterial blood. Our previous [12,13] and current studies explored this still debatable issue to find out the most plausible explanation of the PPG signal taking into account recent findings obtained with IPPG systems. Successful solving of this issue should allow advanced mapping of the microcirculation parameters of human skin.

Observation of the largest PPG signal at the green illumination [7,8,11], despite the very small penetration depth of the light at this wavelength [2,19], and a significant increase in the green light induced PPG signal by a skin-glass contact [12,13], forced us to propose a new model of PPG [13]. According to this model, the main origin of light modulation in PPG is linked to local changes in absorption and light scattering within the capillary bed in the dermis rhythmically compressed by a changeable arterial pressure [13]. The fast increase of the peripheral arterial pressure originating from the blood stroke during the systole provides the force that leads to enlargement of the artery cross-section. This enlargement results in the deformation of adjacent tissues containing blood, such as capillaries and superficial venules. Importantly, the blood volume in capillaries is almost constant during the single cardiac cycle. Therefore, the compression of the dermis primarily reduces the distance between the capillaries, having little effect on the size of each capillary. The skin is usually rather elastic, and it expands under normal conditions to adjust the tissue deformation, thus having little effect on the density of the capillary bed. However, in our experiments, when under rhythmically changed pressure in blood vessels, the skin is in contact with the glass plate, and the capillary bed will be denser than in the contactless conditions. Red blood cells inside the capillaries act as absorption and scattering centers. Therefore, a local decrease in the distances between the capillaries results in a local increase of light attenuation (and larger amplitude of the PPG waveform). Notably, a change in any other internal pressure affecting the dermis (e.g., slow varying blood volume in veins) should also result in variations of the PPG signal such as a slow drift of the DC level.

Consistent with our hypothesis, we found that the role of elastic deformations of the dermis in formation of the PPG waveform is best presented at green illumination because of its small penetration depth (from 0.3 to 0.8 mm [2,19]), whereas arteries are typically situated deeper than 3 mm below the epidermis [20]. In this paper, we show for the first time that the skin-glass contact also leads to an increase of the PPG amplitude at NIR light (Fig 3). However, in contrast to green light, the role of dermis deformations could be less efficient at NIR light because of its larger penetration depth (1.5–2.5 mm [2,19]) and higher probability to interact with the arterial blood in the reflectance mode PPG. The maximal increase at NIR illumination was observed in the same areas of the palm as at green light (Fig 4a), suggesting that they both originate from similar mechanisms. Moreover, the increase of the light modulation amplitude caused by skin contact with NIR light strongly correlates with that with green light, as shown in Fig 4b. The difference in the PPG-amplitude increase can be attributed to the almost twofold stronger attenuation of the green light in the dermis compared with NIR light [19,21]. Therefore, elastic deformations of the dermis by pulsatile arterial blood have a significant influence on the modulation of NIR light at the heartbeat frequency. In summary, we suggest that the PPG waveform, in contrast with the classical model, follows the beat-to-beat transmural pressure changes in arteries, which affects optical properties of the dermis.

It is worth noting that this study has a clear translation aspect. Further profound understanding of the physiological mechanism of light interaction with living tissue would allow the development of reliable, easy-to-use, and cost-efficient diagnostic methods on the base of imaging plethysmography. In particular, new portable and simple devices capable to assess thermoregulation, to test the status of the skin in aging, and to find other application in clinical dermatology could be developed in near future.

## Conclusions

Our analysis suggests that a significant part of the remitted NIR light is modulated by the local variation in the absorption and light scattering as a result of elastic deformations of the dermis. We conclude that the temporal modulation of the PPG signal results from the variable transmural pressure in superficial arteries. Interestingly, this light modulation mechanism is common for both wavelengths as follows from the strong correlation between signals obtained in green and NIR illuminations. However, our experimental data show that green light is stronger modulated than NIR light in glass-contact conditions. Thus, the practical conclusion is that it is preferable to use green illumination in IPPG systems.

Evidently, the process of light modulation in the dermis requires additional studies, and the currently presented model should be further developed and supplemented. However, it is clear that the elastic deformations of the dermis should be taken into account to explain the origin of the PPG signals.
